# Treatment of recurrent aphthous stomatitis (RAS; aphthae; canker sores) with a barrier forming mouth rinse or topical gel formulation containing hyaluronic acid: a retrospective clinical study

**DOI:** 10.1186/s12903-019-0850-1

**Published:** 2019-07-16

**Authors:** Domenico Dalessandri, Francesca Zotti, Laura Laffranchi, Marco Migliorati, Gaetano Isola, Stefano Bonetti, Luca Visconti

**Affiliations:** 1 School of Dentistry, University of Brescia, Piazzale Spedali Civili 1, 25123 Brescia, Italy; 2 Department of Surgical Sciences, Paediatrics and Gynecology, University of Verona, Policlinico “Giovanni Battista Rossi” Piazzale Ludovico Antonio Scuro, 37134 Verona, Italy; 3 School of Dentistry, University of Genova, Largo Rosanna Benzi 10, 16132 Genoa, Italy; 4 Department of General Surgery and Surgical-Medical Specialities, School of Dentistry, University of Catania, via Plebiscito, 625. Building 15a, 95123 Catania, Italy

**Keywords:** Hyaluronic acid, Recurrent aphthous stomatitis, Barrier forming, Rinse, Topical gel

## Abstract

**Background:**

Use of hyaluronic acid-based products has become a valuable alternative to drug-based approaches in the treatment of recurrent aphthous stomatitis (RAS). The presented study aimed to investigate the effect of a barrier forming hyaluronic acid containing mouth wash or a topical gel formulation on the healing of RAS and patient’s quality of life.

**Methods:**

For this single-center retrospective study, medical records of the Dental School of the University of Brescia were screened for adult and systemically health patients suffering from minor recurrent aphthous stomatitis (RAS) and treated with either a barrier forming, hyaluronic acid containing mouth wash (GUM® AftaClear® rinse) or a topical gel (GUM® AftaClear® gel) in 2015. All patients fulfilling the in−/exclusion criteria and presenting full data sets on lesion diameter, lesion color, as well as pain perception for baseline (day 0) and 4 and 7 days after treatment were enrolled into the presented study.

**Results:**

Out of 60 screened patients, a total of 20 patients treated with the Rinse formulation and 25 treated with the Gel formulation were eligible for the enrollment into this study. Both groups showed equal distribution in patient’s age, sex and presented a similar mean lesion size (3.0 ± 1.0 mm), lesion color distribution as well as pain perception at baseline. All patients showed significant normalization of lesion color, reduction of pain, and lesion dimension within the course of their treatment. After 7 days, the mean percentage of lesion reduction was highly significant for both groups attaining 77.4 ± 30.1% in the Rinse group and 81.2 ± 23.1% in the Gel group with a complete lesion closure obtained in 60 and 56% of the cases, respectively. However, a significant (*p* < 0.05) higher percentage of lesions in the Gel group (72%) compared to the Rinse group (40%) showed an improvement in lesion size already after 3 days.

**Conclusions:**

Within the limitation of retrospective design, it can be concluded that both the barrier forming hyaluronic acid containing mouth rinse as well as the topical gel formulation are effective in the treatment of minor recurrent aphthous stomatitis (RAS), with a trend for an earlier healing onset for the topical Gel formulation.

## Background

Mouth ulcers represent a very common unpleasant oral mucosal disease that can reduce patient’s quality of life, due to the presence of a painful stinging sensation that worsen during daily activities like speaking, eating or even drinking [1]. Causes include physical trauma, radiation, chemical injury, and microbial infection (bacterial, viral, and fungal). However, some ulcerations, such as recurrent aphthous stomatitis (RAS), commonly known as aphthae or canker sores, have uncertain etiology and causes of RAS are not fully elucidated [1]. Nevertheless, they share a similar etiopathology. Commonly observed histopathological changes in the pre-ulcerative stage include infiltration of the epithelium by mononuclear (lymphocytic) cells before an edema, followed by keratinocyte vacuolization and focal vasculitis causing localized swelling. This tumefaction ulcerates and infiltration of neutrophils, lymphocytes, and plasma cells occurs until healing and epithelium regeneration. The progression accompanied by a cell-mediated immune response involves T-cells and promotes tumor necrosis factor alpha (TNF-α) generation by activated macrophages [2].

Although the histopathological progression of the aphthous lesions formation follows a common pattern, triggers vary between individuals and may include nutritional deficiencies, local trauma, stress, hormonal influences, allergies, genetic predisposition or other factors [3]. Most RAS appear on non-keratinizing oral mucosa, which excludes the attached gingiva, the hard palate and the dorsum of the tongue. Still there are less common, more severe forms that may also involve keratinizing epithelial surfaces. Symptoms associated with RAS can range from a minor nuisance, local stinging that can interfere with eating and drinking, whereas, the severe forms may be debilitating, even causing weight loss due to malnutrition [3].

RAS can be classified phenotypically into 3 different types although their classification vary slightly in terms of size and healing time between different authors [3–6]: 1. Minor RAS are the most frequently observed (80%) small, round, clearly defined below commonly below 5 mm in diameter (2–3 mm in average) but painful ulcers that typically heal between 7 and 14 days without scaring. 2. Major RAS that account for about 10% of all RAS are larger (diameter exceeds 10 mm) and deep, can last for 6 weeks or longer, and are affecting both mucosa and keratinized tissues, and frequently heal with scaring. 3. Herpetiform RAS, may look ‘herpetic’ in nature but do not have a viral etiology, are mostly present as multiple small clusters of pinpoint lesions that form large irregular ulcers, and similar to minor RAS, heal within 14 days without scaring.

Therefore, the majority of the RAS will heal within 10–14 days without any complication, and ulceration episodes occur 3–6 times per year [3–5].

Incidence of RAS in the general population is estimated around 25%, with an early onset during adolescence or even childhood [4, 5]. The most frequently employed treatments for RAS are anti-inflammatories, corticosteroids, analgesics and antimicrobial, but also lubricating and healing promoting agents are used [5]. Even more advanced anti-TNF-α treatments have been developed using therapeutic molecules directly targeting TNF-α production and, by that, reducing inflammation an immunological host response. However, such approaches suffer from severe side effects ranging from somnolence to nausea and gastrointestinal symptoms. By consequence, Thalidomide, being the most effective and reliable agent among them, is still limited to short term use considering its side effects. Since RAS conditions can last for several years with reoccurring episodes of ulceration before gradually disappearing, long-term exposure to this medication seems not to be appropriate. [5, 7–10].

To overcome the limitations of short term application and severe side effects, other therapeutics have been investigated for the treatment of RAS, including the use of Vitamine B12 [11], silver nitrate [12] and plant extracts [13, 14] with limited success.

In order to get away from a drug-based treatment approach, topically applied mucosal protectants have been developed aiming to form a temporal physical barrier over the ulcerous lesion protecting it from oral traumas [15]. Such an oral wound patch preventing the detrimental and often painful contact with the milieu of oral cavity and food and beverage components, therefore, reducing pain and fostering the healing process [16]. To maintain the barrier in place, the domiciliary application must be repeated several times during day until resolution of the ulcer. Initial clinical studies using topical gels containing hyaluronic acid have shown promising results, demonstrating a significant reduction of the healing time as well as a relief of pain reached by using physical barriers without any reported side-effects [15–18].

Within this class of treatments, two new topical products containing hyaluronic acid as well as mucoadhesive components have recently been introduced in the market: a gel formulation that can be applied directly on the ulcer (GUM® AftaClear® gel, Etoy, Switzerland) and mouth rinse formulation forming a thin layer in situ after rinsing (GUM® AftaClear® rinse, Etoy, Switzerland). Even if hyaluronic acid efficacy in RAS treatment is well known in the literature, it is also reported that patient compliance and topical agent retention on the oral mucosa play a key role in clinical effectiveness. Hyaluronic acid retention is mediated and influenced by formulation of preparation used to convey this agent on the ulcers area, and therefore it varies not only between different products, but also between different administrations ways, such as topical gel, spray or mouth rinse of the same product. Moreover, patient’s compliance is influenced by single formulation characteristics, such as the easiness of use or the pleasant of both product taste and texture. For all the above mentioned reasons it is always prudent, before a priori accepting the beneficial effects of a new product, to clinically test it.

Therefore to evaluate the performance and safety of these two recently available formulations, a retrospective study has been designed aimed to the collection of clinical data from RAS patients treated at the University of Brescia in Italy during the period of January 1st to December 31st in 2015.

## Methods

### Patient selection

This single-center observational retrospective study was approved from the Ethical Committee of Spedali Civili of Brescia (NP 2544) on 20-12-2016. Data was obtained from files of adult patients suffering from recurrent aphthous stomatitis (RAS) arrived to Dental School of the University of Brescia and recruited for a prospective study that, for organizational reasons, was interrupted before its completion.

Briefly, patients using the GUM® AftaClear® mouth rinse formulation rinsed for 60 s for 3 times per day, after meals, repeating as necessary. Patients using the gel formulation applied around 1 cm of the oral gel for to each ulcer for 3 times per day, after meals, repeating as necessary, while used a medium-bristled manual toothbrush during healing period.

Inclusion criteria were: male and female patients aged 18–60 years, treated during the year 2015 at the Dental School of the University of Brescia; use of GUM® AftaClear® gel or GUM® AftaClear® rinse for aphthous mouth ulcers for at least 7 days; report of recurrent aphthous ulceration onset during the last 6 months before start of aphthous mouth ulcer treatment; and registration into the clinical file from baseline till the end of treatment of an evaluation in terms of ulcer dimension and redness of the lesions at different times, e.g. on first day, after 3 days of treatment and/or after 7 days of treatment. Redness of ulcerous lesions was assessed as follows: stage 6 - intense grey-yellow with red edges (GY-R); stage 5 - intense grey-yellow without red edges (GY); stage 4 - yellow (Y); stage 3 - grey (G); stage 2 - red (R); and stage 1 - normalized (N).

Exclusion criteria were: history of any treatment for cancer in the last 3 months; recurrence of cancer and end stage of it; presence of severe systemic diseases, auto-immune or virus-based oral mucosal diseases; lactation or pregnancy; use of any medication to treat ulcers the previous week before gel or rinse use started or use of local medication or systemic drug during the treatment with gel or rinse.

### Data collection

No specific sample size was calculated for data collection. All patients have been included that started the treatment with either rinse or topical gel formulation in the period from January to December 2015, that met the inclusion/exclusion criteria, and sufficient data could be retrieved from their medical records.

After the inclusion/exclusion criteria assessment, the following information were independently collected by two investigators and compared for exactness:

1. Patient demographics (age, gender).
2. Type of recurrent aphthous stomatitis (RAS): Minor ≥4 mm and < 10 mm, Minor < 4 mm, herpetiform. Major aphthous lesions were excluded.
3. Initial size of each ulcer or, in herpetiform cases, of the biggest one.
4. Type of formulation used for the treatment (Rinse or Gel).
5. Frequency of application per day.
6. Decrease of the lesion dimension after 3 and 7 days (in %).
7. Change of color of the ulcerous lesions was characterized using a six stages scale: stage 6 - intense grey-yellow with red edges (GY-R); stage 5 - intense grey-yellow without red edges (GY); stage 4 - yellow (Y); stage 3 - grey (G); stage 2 - red (R); and stage 1 - normalized (N).
8. Reported Pain intensity on 4 levels (No = 0, low = 1, medium = 2, high = 3) at the treatment beginning, after 3 and 7 days.
9. Number of patients with disturbance while eating and drinking.
10. Number of patients with reported improvement in quality of life.
11. Adverse events and adverse device events and concomitant medication.

Data were recorded in a web-based electronic case report form (CeRF) by the Investigators, after the medical chart check was carried out for each patient. Each investigator entered data via a secure network, with access requiring specific username and password. Data management and statistical evaluation was carried out by a fully blinded external CRO ensuring confidentiality and compliance with applicable data privacy protection laws and regulations.

### Statistical analysis

Standard descriptive statistics were performed. Means ± SD was calculated for all parameters. To determine the statistical significance (^#^*p* < 0.001; *p < 0.05) of changes within groups a non-parametric Wilcoxon signed rank test was used since normal distribution of the difference could not be guaranteed. Still, a parametric paired sample t-test was used to calculate *p*-values within groups.

To test for statistical significant difference between the groups at different time points, a non-parametric Wilcoxon rank sum test was used to calculate *p*-values. *p* < 0.05 was considered to be significant.

The “N-1” Chi-squared test (https://www.medcalc.org/calc/comparison_of_proportions.php) as recommended by Campbell [19] was used to assess significant difference between proportions of percentage of closure between the two groups.

## Results

### Patient population

**Fig. 1:**
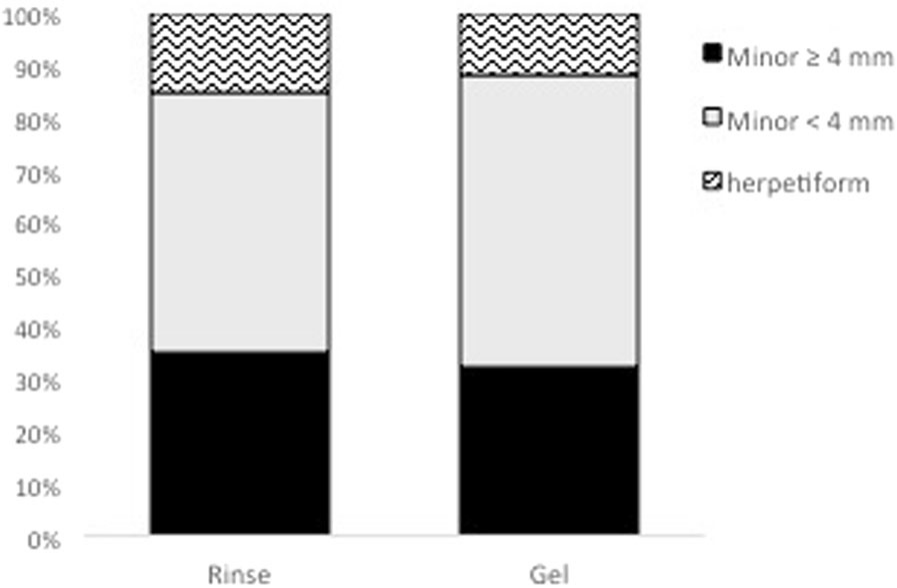
Distribution of recurrent aphthous stomatitis (RAS) types at baseline

**Fig. 2:**
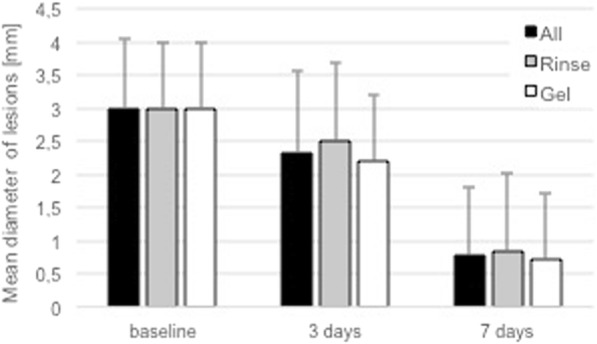
Mean aphthous lesion diameter [mm] and mean percentage of lesion closure [%]

**Fig. 3:**
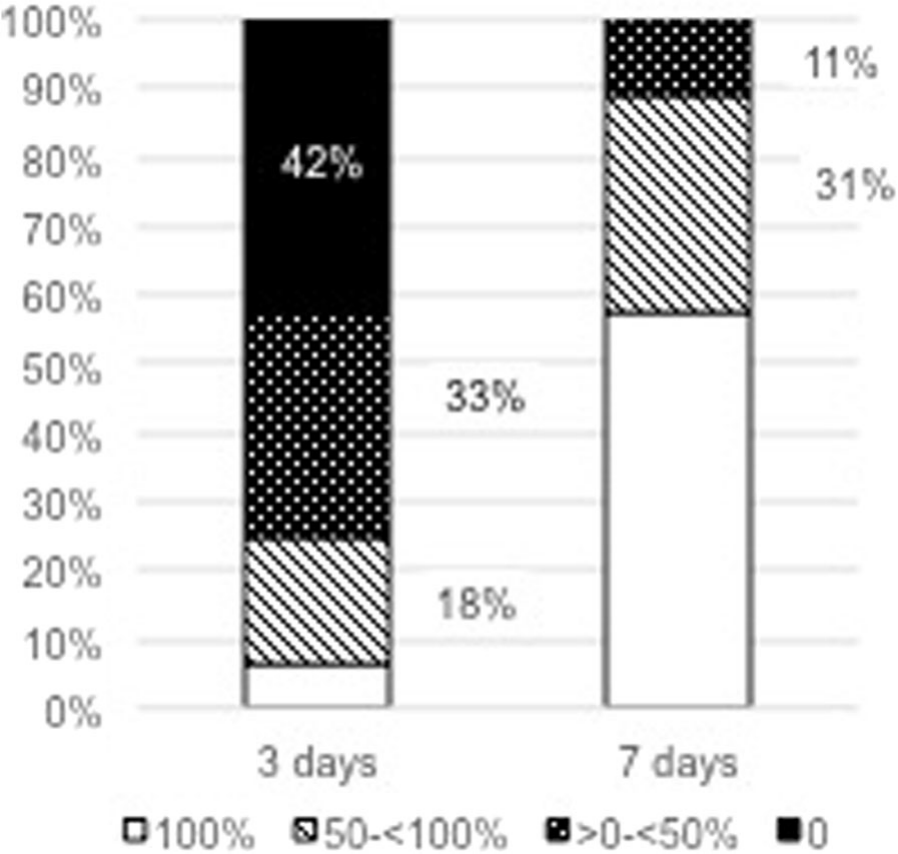
Overall distribution of percentage of lesion closure after 3 and 7 days (all sites)

Out of 60 screened subjects, 45 fulfilled the in/−exclusion criteria and were enrolled into the study. Twenty were treated with rinse and 25 were treated with gel. Twenty-one of the patients were females (Rinse = 11; in the Gel = 10) and 24 were males (9 in the Rinse and 15 in the Gel group). All of them were aged between 24 and 59 years. Mean age of patients treated with the rinse formulation (Rinse) was 45 ± 10 years and 43 ± 12 years for the patients treated with the gel formulation (Gel). The different types of recurrent aphthous stomatitis (RAS) (Minor ≥4 mm and < 10 mm, Minor < 4 mm, herpetiform) were equally distributed between the two groups (Fig. 1), with 35% Minor ≥4 mm and < 10 mm (7), 50% Minor < 4 mm (10), and 15% herpetiform (3) in the Rinse group; and 32% Minor ≥4 mm and < 10 mm (7), 56% Minor < 4 mm (10), and 12% herpetiform (3) in the Gel group. Average lesion size at baseline was equivalent for both groups and all lesions had a grey yellow appearance before the beginning of treatment (Figs. 2 and 3).

Since the baseline, all patients reported pain of different intensity and difficulties in eating and/or drinking.

Most patients did apply the rinse as recommended in the instructions for use, 3 times a day. In detail, the rinse was administered 3 times/day by 18 (90%) patients and 5 times/day by 2 (10%) patients; the gel was administered 3 times/day by 22 (88%) patients and 5 times/day by 3 (12%) patients. Neither adverse events (AE) nor concomitant medications were reported in patient’s files during the 7-day treatment, assuring on the lack of interferences with the healing process, or any of study assessments such as pain perception.

### Reduction of mean aphthous lesion diameter and percentage of lesion closure

Both groups presented a similar distribution of RAS types and identical mean lesion diameter of 3.0 ± 1.0 mm (Fig. 2; Table 1) at baseline.

**Table 1:** Mean lesion diameter and percentage of lesion closure after 3 and 7 days

	n	Lesion diameter [mm]			Percentage of closure [%]		
		baseline	3 days	7 days	baseline	3 days	7 days
All	45	3.0 ± 1.0	2.3 ± 1.2	0.8 ± 1.0	0	25.6 ± 28.4	79.5 ± 26.2
*p*-value		*–*	*1.17E-8* ^#^	*5.94-E24* ^#^	–	–	–
Rinse	20	3.0 ± 1.0	2.5 ± 1.2	0.9 ± 1.2	0	17.9 ± 26.8	77.4 ± 30.1
*p*-value		*–*	*0.0442* ^#^	*3.29E-10* ^#^	–	–	–
Gel	25	3.0 ± 1.1	2.2 ± 1.2	0.7 ± 0.9	0	31.7 ± 28.7	81.2 ± 23.1
*p*-value		*–*	*3.65E-07* ^#^	*8.88E-15* ^#^	–	–	–

^#^*p* < 0.001; *p < 0.05 for Wilcoxon Signed Rank Test; *p*-value from paired sample t-test

All sites showed a significant improvement of lesion dimension during the observed treatment period. Overall percentage of mean lesion closure in 45 patients was significantly increased over the observed period averaging to 25.6% ± 28.4% at day 3 and 79.5% ± 26.2% at day 7, respectively. In total, 3 patients (7%) showed a complete closure of the lesion after 3 days and 26 (58%) presented a complete healing after 7 days (Table 2).

**Table 2:** Distribution of lesion closure grade within the two treatment groups after 3 and 7 days

Grade of closure	3 days			7 days		
	All (*n* = 45)	Rinse (*n* = 20)	Gel (*n* = 25)	All (*n* = 45)	Rinse (*n* = 20)	Gel (*n* = 25)
100%	7%	5%	8%	58%	60%	56%
50- < 100%	18%	10%	24%	31%	20%	40%
> 0- < 50%	33%	25%	40%	11%	20%	4%
0	42%	60%	28%	0%	0%	0%

**Fig. 4:**
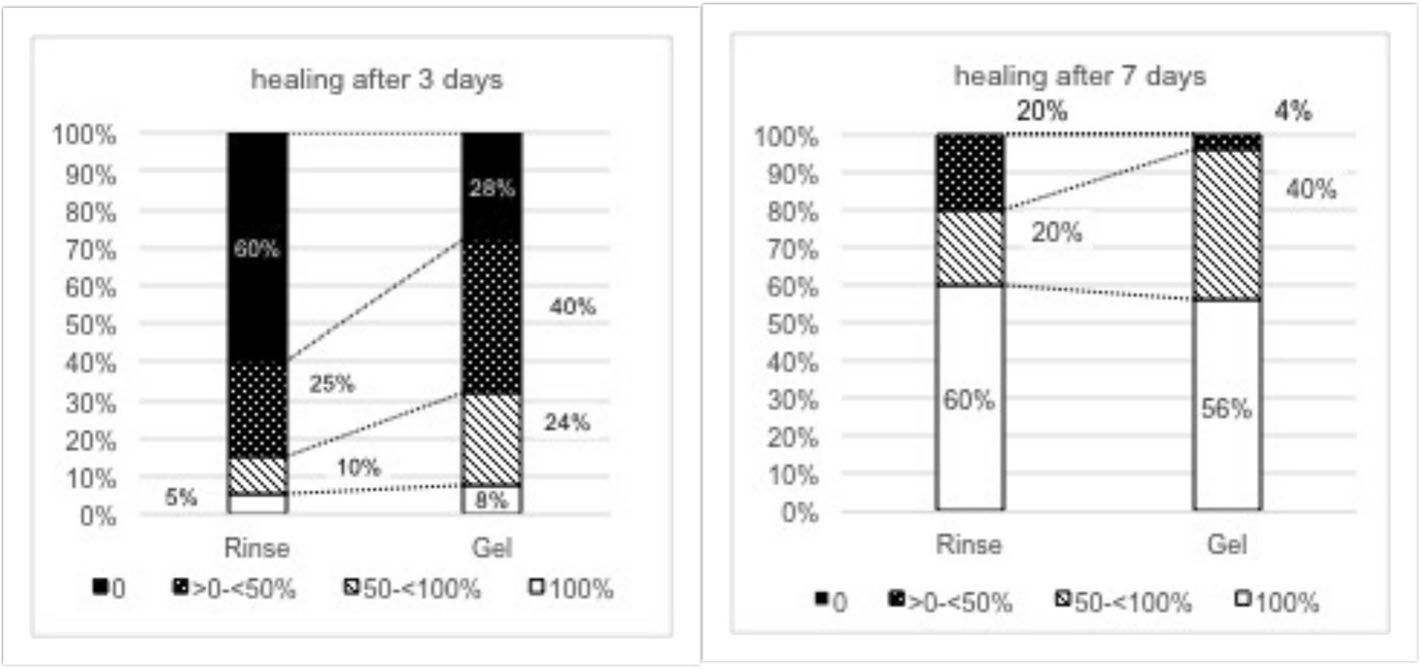
Degree of lesion closure for Rinse and Gel group after 3 and 7 days

Looking at the individual treatment groups separately, the percentage of mean lesion closure was significant (17.9 ± 26.8%, *p* = 0.0442) for the Rinse group and highly significant (31.7 ± 28.7, *p* = 0.0000000365) for the Gel group after 3 days (Table 1). After 7 days, the mean percentage of lesion closure was highly significant for both groups compared to baseline, attaining 77.4 ± 30.1% in the Rinse group and 81.2 ± 23.1% in the Gel group (Table 1). In principle, no statistically significant differences (*p* < 0.05) could be observed between the two treatment groups regarding the mean percentage of lesion closure or the number of completely healed sites (100% healing – Fig. 4) at any observation time-point.

Although, neither the overall mean percentage of lesion closure nor the percentage of sites with complete lesion closures did reveal significant differences between the two groups. Regarding the percentage of sites showed healing progression, a significant difference could be observed. At day 3, 40% of all RAS treated with the Rinse formulation did show a reduction of their lesion diameter (> 0% healing), whereas already 72% of all lesion treated with the Gel formulation showed improvements from baseline compared with the same period (Table 2; Fig. 4). This difference was statistically significant (*p* < 0.0327). After 7 days, a certain delay in healing in the Rinse group compared to the Gel group was still evident when looking at the percentage of RAS with percentage of lesion closure of less than 50% (Fig. 4–0% and > 0- < 50%). Still 20% of the RAS in the Rinse group, but only 4% in the Gel group, showed a percentage of lesion closure inferior to 50% after 7 days. However, this difference did not reach statistical significance (*p* = 0.0933) after 7 days.

Overall, neither the age nor the gender of the patient, nor the type or baseline size of RAS had a significant influence on the lesion healing progression in this study.

### Appearance of aphthous lesions at baseline, 3 days, and 7 days (color)

**Fig. 5:**
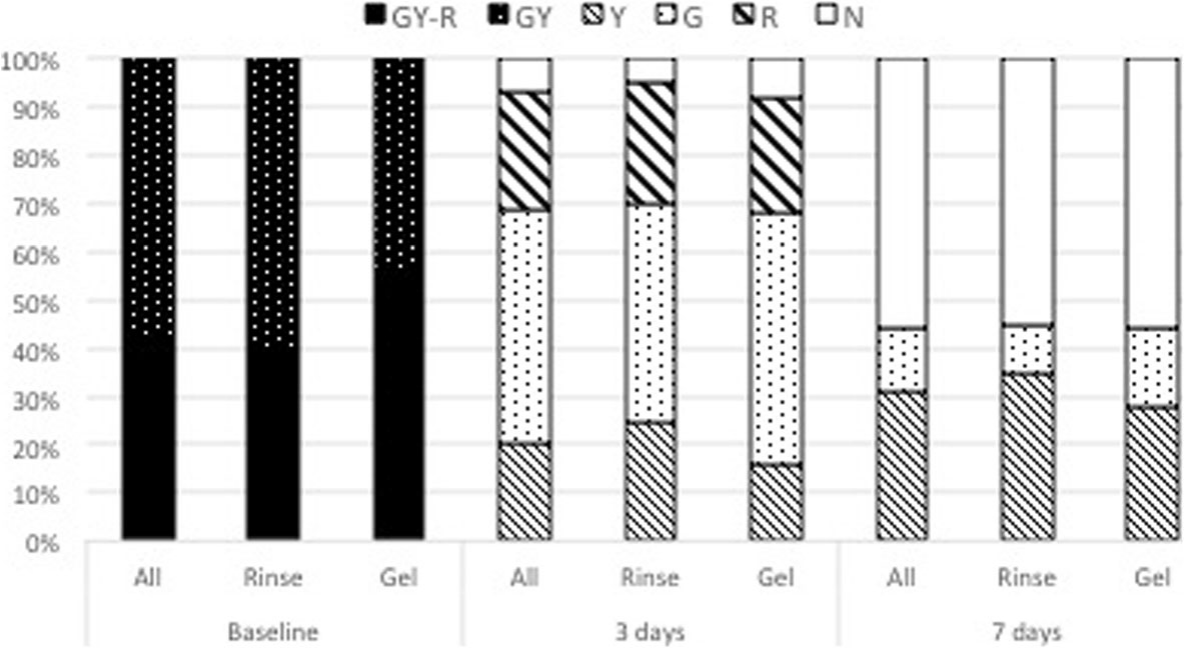
Distribution of lesion color grades at baseline and after 3 and 7 days

Coloration of the aphthous lesions can give additional information about the healing progression. The appearance of the aphthous lesions were graded from intense grey-yellow with red edges (GY-R) to faded without red edges (GY), Red color (R), Yellow (Y), Grey (G), and normal appearance (N). Reduction of redness and adaptation of lesion color to surrounding mucosa was judged as a sign for healing. At baseline, all lesions displayed an intense grey-yellow color, whereas, 40% in the Rinse group and 56% in the Gel group displayed additional red edges. This indicated that all lesions represented fresh, unhealed aphthous ulcers (Fig. 5). There was no statistical difference between the two groups in terms of appearance at baseline.

Healing progression, assessed by reduction of lesion redness, and change in coloration towards a more normal appearance were noticed in all patients, with first positive results already visible after 3 days of treatment. Forty-two subjects (93.30%) out of all treated 45 patients (Rinse and Gel) showed already a color improvement of lesion at day 3. In both groups, lesion color changed from intense grey-yellow appearance with red edges (GY-R) or faded grey-yellow appearance without red edges (GY) to grey (G), yellow (Y), red (R) or normalized (N) appearance after 3 days. Only 3 patients already presented a normal lesion color after 3 days, however lesions appearance was judged normal in a total of 25 patients (55.55%) after 7 days. Changes of coloration over time were statistically significant (*p* < 0.05) for both treatment groups. However, no statistical differences in the composition of lesion colors was observed between the two groups at any time-point.

### Improvement of quality of life, changes in pain intensity, and disturbance when eating or drinking

All except 1 patient (Gel group) stated their quality of life has improved during the treatment with the Rinse and the Gel formulation until day 7. The diameter of baseline lesion of respective patient was rather large (diameter of 4 mm) and it was only reduced to 50% from its initial size until the end of treatment, where a lesion diameter of 2 mm was still detectable. Although he did not report on any disturbance while eating or drinking and at day 7 he still score his pain as medium level. Unfortunately, no further data was available to better explain his subjective assessment.

At baseline, all 45 patients reported pain and stinging sensation with different intensity, furthermore issues with either drinking or eating or both were highlighted. Mean perception of pain intensity (none = 0, low = 1, medium = 2, high = 3) in the two groups was similar at baseline (Rinse = 1.90 ± 0.72; Gel = 1.92 ± 0.81) and significantly decreased at day 3 (Rinse = 1.10 ± 0.85; Gel = 1.16 ± 0.85) and day 7 (Rinse =0.45 ± 0.76; Gel = 0.40 ± 0.65), respectively (Table 3).

**Table 3:** Mean reported pain intensity at baseline and after 3 days and 7 days

	Mean pain score (± SD)		
	Baseline	3 days	7 days
Rinse	1.90 ± 0.72	1.10 ± 0.85	0.45 ± 0.76
*p*-value	*–*	*4.6E-08#*	*9.8E-11#*
Gel	1.92 ± 0.81	1.16 ± 0.85	0.40 ± 0.65
*p*-value	*–*	*1.7E-07#*	*2.5E-12#*

^#^*p* < 0.001,*p < 0.05 for Wilcoxon Signed Rank Test; *p*-value from paired sample t-test

There were no statistically significant differences in terms of reported mean pain intensity (*p* < 0.05) between the two groups at any time-point.

**Fig. 6:**
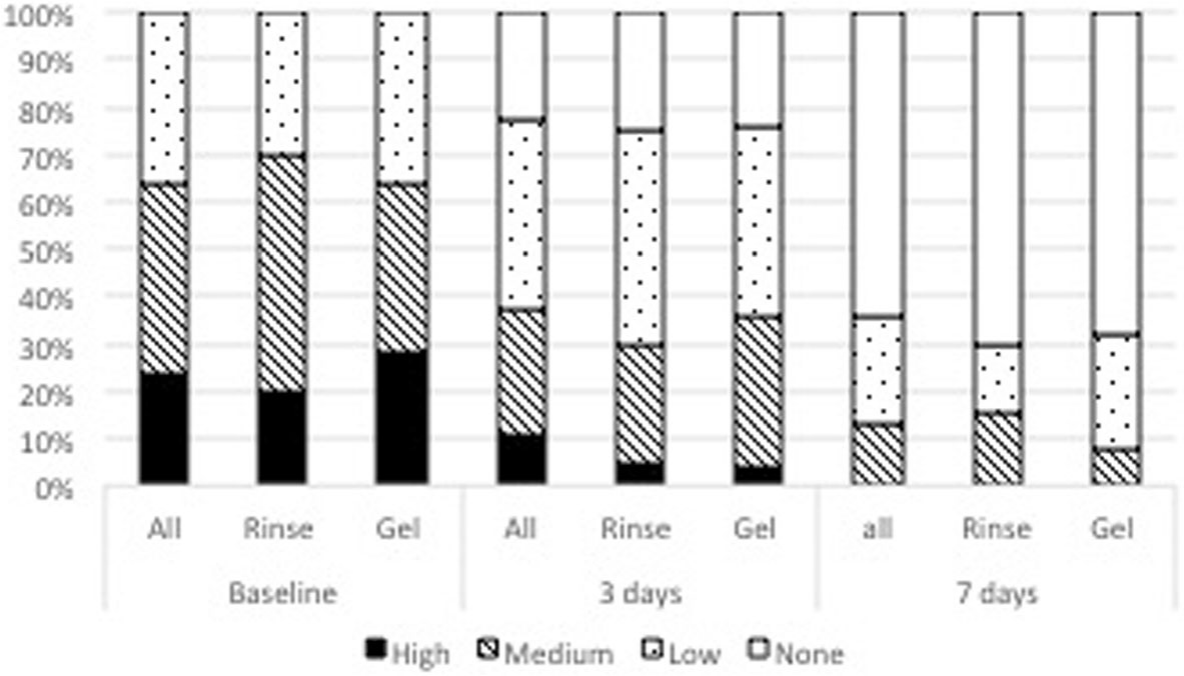
Distribution of reported pain intensity at baseline and after 3 and 7 days

A total of 5 patients (35%) in the Rinse group and 6 patients (24%) in the Gel Group reported no pain after 3 days, whereas 12 patients (70%) in the Rinse group and 17 patients (68%) in the Gel group were free of pain after 7 days (Fig. 6).

**Fig. 7:**
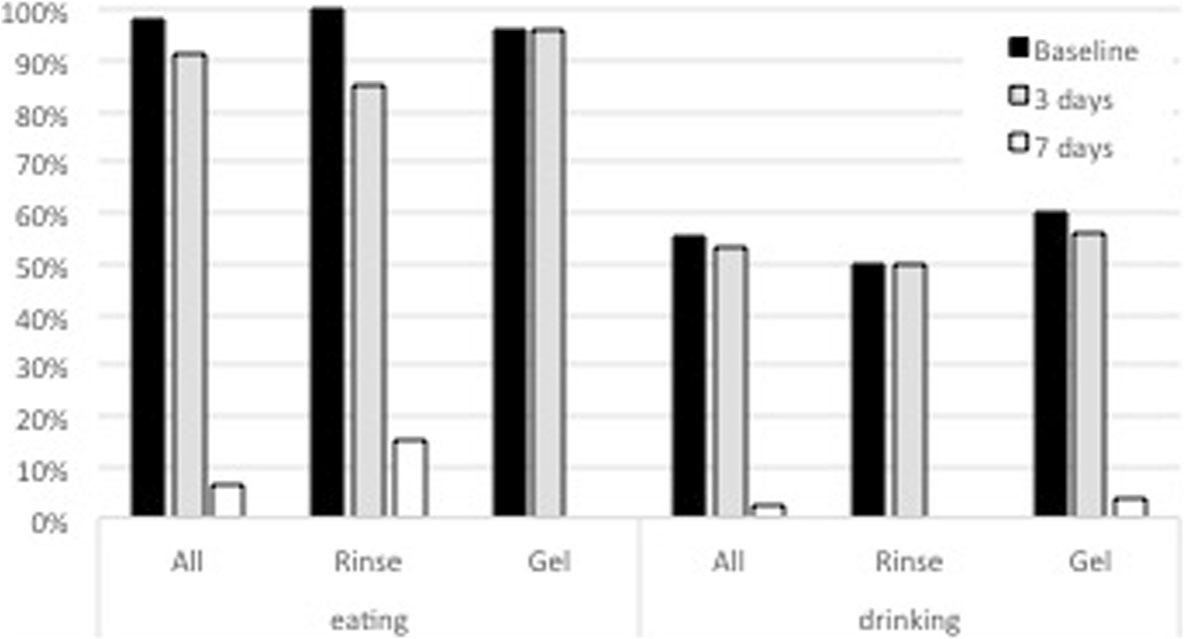
Percentage of patients with eating and drinking disturbance at baseline and after 3 and 7 days

Independently from their pain intensity, all patients (100%) in the Rinse group reported a disturbance when eating and 50% when drinking at baseline (Fig. 7). Likewise, patients with problems in eating represented 96% and those with problems in drinking represented 60% of patients in the Gel group at baseline. Both these disturbances while eating and drinking did not improve significantly at day 3, irrespective of the treatment (Fig. 7). However, the reduction of patients reporting a disturbance when eating or drinking was highly significant for both groups after 7 days compared to baseline as well as the 3-day time point (*p* < 0.0001). No significant difference between the two treatment groups could be observed at any of the time-points (Table 4).

**Table 4:** Distribution of reported pain intensity within the two treatment groups (Rinse, Gel)

	Baseline		3 days		7 days	
	Rinse (*n* = 20)	Gel (*n* = 25)	Rinse (*n* = 20)	Gel (*n* = 25)	Rinse (*n* = 20)	Gel (*n* = 25)
High	4 (20%)	7 (28%)	1 (5%)	4 (20%)	0 (0%)	0 (0%)
Medium	10 (50%)	9 (36%)	5 (25%)	8 (32%)	4 (15%)	2 (8%)
Low	8 (30%)	9 (36%)	9 (45%)	10 (40%)	4 (15%)	6 (24%)
None	0 (0%)	0 (0%)	5 (25%)	6 (24%)	12 (70%)	17 (68%)

After 7 days only 7% of the patients still reported some disturbance when eating, whereas, only 2% still felt pain when drinking (Fig. 6).

## Discussion

The recurrent aphthous stomatitis (RAS) is the most common form of recurrent oral ulcers. RAS is characterized by recurring painful lesions in the mouth with a round or ovoid appearance and inflammatory halos. Individual aphthous ulcers may be morphologically classified in 3 different types. Minor RAS are shallow lesions in mucosa with diameters up to 5 mm and heal over 7 to 14 days. Major RAS exceed 10 mm, are deep, can affect also the keratinized mucosa, and heal over several weeks, sometimes with scaring. The herpetiform RAS are commonly 2–3 mm in diameter, however patterns fusing into bigger ulcers and varying in diameter and depth as well in the healing time, generally reported between 10 and 14 days, are described [1, 5, 16]. In order to investigate the performance of a barrier forming, hyaluronic acid-based gel and mouth rinse formulation in the treatment of active RAS, through the assessment of lesion and pain reduction, only patients with minor RAS and herpetiform types with a lesion diameter between 2 and 5 mm were considered for this retrospective study.

Results reported in this study corroborate the trend towards a faster or earlier healing of the lesions treated with the topical Gel is expected, even if both groups displayed a very similar mean lesion reduction after 7 days.

In this study patients could choose freely between the Rinse or Gel formulation, based on their individual preference irrespective of their initial situation. Both formulations showed to be very effective and indicated in treatment of minor and herpetiform RAS in general. The presented clinical data seem to indicate a preference towards the topically applied Gel in the most acute phase of ulceration (first 3 days) and in larger ulcer diameters. Moreover, the Rinse formulation seems to be the ideal application form to prevent formation of RAS before an aphthous lesion is visible, due to its ability to reach the entire oral cavity. A daily application could, therefore, reduce the frequency of recurrence of RAS. However, the effect on the recurrence frequency was not investigated in this study and it would need to be investigated in prospective design.

Regarding methodological aspects, limitation of the presented retrospective study is lack of a negative control, placebo or sham group. Moreover prospective randomized controlled trial based on these results might be useful in order to test effects of these treatments on a negative control, placebo, or sham group. Nevertheless, feasibility to compare this database of signs and symptoms to those in up-to-date literature might be a great opportunity both to consolidate existing knowledge and to address future clinical studies in inclusion and exclusion criteria designing. RAS is a quite widespread pathological condition in population, so that clinicians and general dental practitioners could take advantage from this study also to manage the anamnestic information of their patients and to collect a proper and workable medical file.

Still, sufficient publications reporting on the treatment of RAS with similar or drug-based approaches and presenting data on equivalent clinical endpoints (percentage of lesion reduction and pain perception) exist and allow a comparison to our data.

Both, results here obtained on percentage of lesion closure as well as on percentage of completely healed site at day 3 and 7 were comparable or superior to data reported from other groups using similar topically applied barrier-forming protective agents in minor RAS types of around 3 mm diameter. For example, application of a film-forming triamcinolone acetonide (TA) pomade or a triester glycerol oxide gel (TGO) resulted in a 38 and 46% of mean lesion reduction after 4 days, and 70 and 77% after 7 days, respectively [20]. Although the results from the 3 days’ time points in our study cannot be compared to the 4 days’ time point in their study, after 7 days, percentage of lesion closure was found to be equal or inferior to our results. Similar findings were reported in a study including 16 patients with minor RAS of around 3 mm diameter treated with a topical application of a 0.2% hyaluronic acid gel (Gengigel®) [16]. Although the group assessed healing after 14 days only, the maximum ulcer size was found to be reduced by 62%, number of ulcers per patient was reduced only by 50% during this elongated period. Moreover, 18.8% of the patients still reported to feel no improvement after 14 days.

Solely data from a study investigating the topical application of anti-inflammatory and anti-bacterial drugs showed slightly superior results in terms of mean lesion reduction with 75 and 54% of reduction after 4 days and 95 and 80% reduction after 7 days, for a 2% quercetin loaded hydroxyethylcellulose gel and its control a benzylamine hydrochloride-based mouth rinse, respectively. Although no patients in the quercetin group reported any side effects, 8 patients applying the benzylamine hydrochloride complained of a stinging sensation after rinsing. Considering the low risk profile of a drug-free formulation compared to the risks associated with the daily application of an anti-bacterial and anti-inflammatory drug, the slightly faster healing reported, probably also related to a slightly different measurement and study population, seems not to outweigh the possible risks.

Our results showed percentage of complete lesion closure equivalent and even superior to the results reported for treatment with 2% Quercetin or benzylamine hydrochloride. Only 60% of the lesion in the quercetin gel group and only 35% in the benzylamide hydrochloride group reached full closure after 7 days. [21]

In our study assessment of lesion color changes during the treatment supported an early improvement of the aphthous lesions (Fig. 7), moreover relationship between reported pain and changes of diameter and lesion color was clearly detected.

Some comparison with following reported clinical data can be drawn, even if in our study a 4-level pain scale (none, low, medium, high) instead of a Visual Analog Scale (VAS) was used to assess pain. Ofluoglu and co-workers [18, 20] reported that after topical application of a pomade formulation, a reduction to a normal pain level (healed = > 95% VAS reduction) was achieved in only 1.9% in the TA group, 8.9% in the TGO group, and 2% in the placebo after 6 days of treatment. The study investigated another hyaluronic acid based topical formulation (Gengigel®) reported a VAS score reduction by 65% after 14 days [16]. Furthermore, a total of 18.8% patients reported no improvement of their situation after 14 days, which was likely related to their pain assessment. Similar findings were reported for another hyaluronic acid gel formulation test showing continues pain reduction from day 0 to day 4 and 7 compared to a pomade formulation [18]. In our study only 2% of all treated patients reported to suffer from disturbances while drinking after 7 days.

## Conclusions

Within the limitation of the retrospective design of the present study and based on the comparison to the published literature, it can be concluded that both these two barrier-forming hyaluronic acid based mouth rinse (Rinse) and topical gel formulation (Gel) are effective in the treatment of minor and herpetiform recurrent aphthous stomatitis (RAS), with a trend for faster healing onset when the topical Gel formulation is applied. Further prospective randomized clinical studies are necessary to assess the reduction of healing time compared to a control group and to investigate the effect of the Rinse formulation on the frequency of recurrence of RAS.

## Data Availability

The datasets used and/or analyzed during the current study are available from the corresponding author on reasonable request.
